# Construction and EST sequencing of full-length, drought stress cDNA libraries for common beans (*Phaseolus vulgaris *L.)

**DOI:** 10.1186/1471-2229-11-171

**Published:** 2011-11-25

**Authors:** Matthew W Blair, Andrea C Fernandez, Manabu Ishitani, Danilo Moreta, Motoaki Seki, Sarah Ayling, Kazuo Shinozaki

**Affiliations:** 1 Bean Program and Biotechnology Unit, International Center for Tropical Agriculture (CIAT), A.A. 6713, Cali, Colombia; 2 Plant Genomic Network Research Team and Director, RIKEN Plant Science Center, 1-7-22 Suehiro-cho, Tsurumi-ku, Yokohama, 230-0045, Japan

## Abstract

**Background:**

Common bean is an important legume crop with only a moderate number of short expressed sequence tags (ESTs) made with traditional methods. The goal of this research was to use full-length cDNA technology to develop ESTs that would overlap with the beginning of open reading frames and therefore be useful for gene annotation of genomic sequences. The library was also constructed to represent genes expressed under drought, low soil phosphorus and high soil aluminum toxicity. We also undertook comparisons of the full-length cDNA library to two previous non-full clone EST sets for common bean.

**Results:**

Two full-length cDNA libraries were constructed: one for the drought tolerant Mesoamerican genotype BAT477 and the other one for the acid-soil tolerant Andean genotype G19833 which has been selected for genome sequencing. Plants were grown in three soil types using deep rooting cylinders subjected to drought and non-drought stress and tissues were collected from both roots and above ground parts. A total of 20,000 clones were selected robotically, half from each library. Then, nearly 10,000 clones from the G19833 library were sequenced with an average read length of 850 nucleotides. A total of 4,219 unigenes were identified consisting of 2,981 contigs and 1,238 singletons. These were functionally annotated with gene ontology terms and placed into KEGG pathways. Compared to other EST sequencing efforts in common bean, about half of the sequences were novel or represented the 5' ends of known genes.

**Conclusions:**

The present full-length cDNA libraries add to the technological toolbox available for common bean and our sequencing of these clones substantially increases the number of unique EST sequences available for the common bean genome. All of this should be useful for both functional gene annotation, analysis of splice site variants and intron/exon boundary determination by comparison to soybean genes or with common bean whole-genome sequences. In addition the library has a large number of transcription factors and will be interesting for discovery and validation of drought or abiotic stress related genes in common bean.

## Background

The legume family is the second most important crop family as a human food source after cereals and in addition provides scores of other products including fodder and feedstock, valuable timber, vegetable oil, bio-fuels, important medicines and even poisons [1]. Legumes are unequalled for stabilization and reforestation of degraded land due to their ability to fix nitrogen, compete with other plants, repel herbivory and grow on acid soils in a range of environments [2]. Many legumes are major elements of international trade because they are high value and a source of protein, calories and oil. Within the legume family, common bean (*Phaseolus vulgaris*) is the most important crop for direct human consumption and is third in overall production after soybean (*Glycine max *L.) and peanuts (*Arachis hypogea *L.). However, unlike these species, beans are primarily grown on small- to medium-scale farms and are not used for industrial processing [3].

Expressed sequence tags (ESTs) are partial sequences of transcribed genes and represent gene expression in different tissues and often different genotypes depending on the plant treatment and development stage at which the mRNA was extracted [4]. ESTs are known to be derived from transcribed mRNA which is cloned into cDNA libraries which are then sequence *en masse*. Therefore, a large effort has gone into constructing many different cDNA libraries for major legume crops such as soybean [5] and model legume species such as *Lotus japonicus *[6] and barrel medic, *Medicago truncatula *[7].

The number of ESTs found for all plant species now is over 21 million sequences. For the legumes a total of over 3 million sequences have been generated with the largest numbers in soybean (1.5 million) and the model legumes barrel medic (280,000) and lotus (242,000). This compares to over 6 million sequences in the *Gramineae *and nearly 3 million in the *Brassicaceae*.

Comprehensive libraries have been made for rice and *Arabidopsis thaliana *for example [8]. Among the legumes, relatively fewer ESTs are found for the crop legumes than in the model legumes and soybean. Among the more minor legume crops, only a recent effort in cowpea (*Vigna unguiculata*) by Muchero et al. [9] nears the threshold of 200,000 total ESTs while common bean has about half that.

In common bean, there have been very few large scale efforts at cDNA cloning or EST sequencing and the current number of ESTs is 114,139 as of December 2010. Preparation of ESTs for common bean began with moderate numbers of GenBank entries by groups from CIAT, UNESP and UNAM [10-12] organizations in Colombia, Brazil and Mexico, respectively, showing the importance of this crop to Latin America.

Additional ESTs have been sequenced or analyzed in US universities such as Univ. of Minnesota [13] and Univ. of Missouri [14]. Among these studies the first medium sized collections by Melotto et al. [11] and Ramírez et al. [12] consisted of 5,243 and 15,333 ESTs or unigenes, respectively. However, these represented EST sequencing of three and five different cDNA libraries, respectively.

The tissues sampled in common bean have represented mainly disease-infected seedling tissue for the set of libraries from Melotto et al. [11] and then a range of tissues from nodules and nodulated roots to leaves and pods for Ramírez et al. [12]. Since then there has been the publication of one large scale EST collection of 37,919 un-trimmed ESTs by Thibivilliers et al. [14] from beans infected with rust (*Uromyces appendiculatus*) and one additional set of ESTs from two root libraries [15]. In addition, a large number 391,150 ESTs have been developed for the suspensor cells of the related species *P. coccineus *by UCLA. Finally, a Canadian group at the Univ. of Saskatchewan has sequenced 10,272 ESTs from *P. angustissimus*, another relative of common bean.

Among other tropical legumes, pigeonpea (*Cajanus cajan *L.), has had an EST project of around 10,000 sequences [16] which are of interest due to the close relationship with common bean and its adaptation to the same dry to sub-humid conditions beans face. Cultivated peanut, *Arachis hypogea*, with 86,935 ESTs plus two ancestral species of peanut with around 32,000 ESTs each are the only other tropical legumes that have been emphasized.

Of these EST collections, only one collection from Ramírez et al. [12] and another rom Blair et al. [15] has represented tolerance to abiotic stresses so far with both research groups emphasizing genes expressed under low phosphorus conditions in roots However, some efforts have been made to evaluate metabolic pathways and clone transcription factors [17] or to sequence differentially expressed cDNAs from drought-treated tissues [18,19]. Therefore, there is a need for additional EST sequencing in common bean and other tropical legumes especially for tissues affected by the drought and soil or weather stresses that are very important issues for productivity of these crops [20].

Among the legumes and for common beans in particular, one aspect of transcriptome analysis and EST sequencing that has been missing is the cloning of full-length cDNA clones. This technology, as first described by Seki et al. [21] and Carnici et al. [22], consists in capture of mRNA through their 5' caps and stabilization of the full transcript during ligation into an appropriate vector and during reverse transcription from the poly A tail [23]. Full-length cDNA libraries have been made for a large range of arabidopsis tissues [24,25] and for several starch-rich crops [26,27] but fewer for legumes, except for soybean [28].

Full length cDNA libraries are extremely useful for analysis of the transcriptome and for comparative genomics and genome sequence validation given that they represent entire transcription units rather than partial gene sequences like most other cDNA libraries [24]. They are especially valuable in that they uncover the transcriptional start site for most genes and EST sequencing of their 5'ends uncovers the un-translated region and methionine-encoding, ATG codon, translational start signal. They can then be used along with non-full length cDNA sequenced clones to cover entire gene sequences allowing scientists to determine where the open reading frame starts and ends and anchoring all this information to genomic sequences.

These characteristics give full-length cDNA sequences essential roles in discovering alternative splicing patterns and promoter regions [23]. In some cases, full length cDNA clones have been used to construct microarrays to characterize the binding of transcription factors to promoter elements within the 5' UTRs of genes [25]. Full length cDNAs also have utility in functional and physical analysis of protein activity and structure through their use as expression vectors as reviewed in [23]. Several examples exist of 3D crystal structure being determined through the use of these clones [29-32].

In addition, full length cDNA clones have a role in characterizing gene structure in different species. For example their 5' and 3'sequences can be used to compare GC content and folding capacity in 5'UTR (un-translated regions) versus ORF (open reading frames) and 3'UTR regions [27].

Finally, as with other sorts of ESTs, full-length cDNA clone sequencing can be used to develop many types of genetic markers including simple sequence repeats (SSRs) which tend to be in greater supply in 5'UTR sequences, single nucleotide polymorphisms (SNPs) especially for different parts of ORFs [33,34]. It is important to use standard genotypes such as those from genome sequencing efforts in the construction of full length cDNA libraries as the genome to gene comparisons become more straightforward when this occurs. In summary, full length cDNA technology can be very important for gene annotation, for sequencing of the transcriptome and for comparative genomics

The objectives of this research, therefore, were to make full-length cDNA libraries that would be useful for gene discovery in common bean, genome annotation of the sequenced genotypes and for an understanding of abiotic stress tolerance in the crop. Multiple treatments were sampled including unstressed, drought, low phosphorus and aluminum stressed plants so as to enhance the activation of the transcriptome machinery and naturally normalize the sampling of mRNAs. Furthermore, two genotypes were used in this initial fl-cDNA library construction, one known to be drought tolerant (BAT477) and the other which is the subject of full-length genomic sequencing (G19833). A total of nearly 10,000 ESTs were generated from the second library to show the utility of this technique in determining gene structure.

This EST sequencing project was performed as part of a breeding project to discover molecular markers in common beans for marginal areas of Sub-Saharan Africa and the process of marker discovery from full-length cDNA sequences is discussed. We also aimed to compare the ESTs from the full-length cDNA library to two previous large EST sets for common bean and show the advantages this technology has for genomic tool development in this less-well studied species.

## Methods

### Plant material

Two elite common bean genotypes were selected based on their attributes for stress resistance and use in genomics studies. First, the Mesoamerican gene pool advanced lines BAT 477 was selected based on its deep rooting ability and known drought tolerance [35], and second, the Andean gene pool genotype G19833 was selected based on resistance to both Al-toxicity and low phosphorus soil stresses [36,37]. This latter genotype has been selected for whole genome sequencing based on the physical map made by Schlueter et al. [38] and refined by Cordoba et al. [39]. The DOR364 × G19833 mapping population described in Blair et al. [40] has also been used for determining the location of interesting genes such as those for red seed color [41] and recessive resistance to BCMV [42] as well as for QTL for nutritional traits [43].

### Treatments, experimental conditions and sampling times

The genotypes were subjected to drought and irrigated (control) conditions as main treatments. Three types of soils with specific properties were collected at different locations including Palmira (highly compacted), Darién (low P content), and Quilichao (high Al content) for a total of six treatments as shown in Additional file 1. The experiment was established under greenhouse conditions using plastic PVC-tubes of 0.8 m inserted in non-translucent sleeves and filled with the specific soil plus a 2:1 soil: sand ratio. The irrigation was stopped at 10 days after seed germination to simulate natural drought stress under all drought treatments. In a split plot design with 2 replicates, a control treatment was normally irrigated throughout the experiment for each soil type. Aerial and root tissues were harvested, washed and frozen immediately with liquid nitrogen for the subsequent total RNA isolations. Harvests of tissues were performed at five-day intervals until reaching 35 days of drought period and sampling from each of the development stages of the plants: seedlings (cotyledons and shoots), growing stage (leaves, stems, shoots and roots), and reproductive stage (flowers and small pods). Roots were carefully obtained by washing away the sand-soil mixture with a light stream of water and then rinsing in a plastic tub. Tissues of the irrigated control were only collected at 15, 30, and 45 days after germination, which were representative of the stages of growing and flowering (see Additional file 2 for explanation of the time course for tissue harvest and for a photograph of the deep root, cylinder culture system).

### Total RNA isolations

Frozen tissues were ground mechanically to a fine powder using liquid nitrogen. Aerial and root tissues with their corresponding treatments and sampling times were processed separately. Total RNA isolations were carried out using the TRIzol^® ^reagent (Invitrogen, Cat. # 15596-018) and following the manufacturer guidelines. Total RNA pellets were re-suspended in RNAse-free water and quantified by spectrophotometry. After quantification each the amount of RNA obtained from each sampling time for the drought and irrigated treatments were pooled separately within each target genotype. Total RNA from aerial and root tissues were also pooled separately. RNA quality was determined through denaturing agarose gels (1.5%) containing formaldehyde and stained with ethidium bromide. Two different primer tags for each genotype, one specific for BAT477 and one specific for G19833 derived mRNAs were used to identify the genotype of the isolated cDNA clones.

### Library construction and EST sequencing

Library construction was performed at the RIKEN institute of Yokohama, Japan with the following abbreviated method using pooled mRNAs from each genotype in separate reactions: Poly (A)^+ ^RNA was prepared with the a μMACS mRNA Isolation Kit (Miltenyi Biotec) following the protocol of the manual. A full-length cDNA library was constructed from the biotinylated poly (A)^+ ^RNA using the CAP trapper method and trehalose-thermoactivated reverse transcriptase. The resultant double-stranded cDNAs were digested with *Bam*HI and *Xho*I, and ligated into the *Bam*HI and *Sal*I sites of a Lambda-based pFLCIII-cDNA vector [21]. During the first strand cDNA synthesis an individualized tag primer sequence was incorporated into the libraries for each genotype: namely 5'-CTGATACG-3' for BAT477 and 5'-GTCATACG-3' for G19833 identified by its placement between the poly A tail and a *Sfi*I (GGCCNNN·NGGCC) site.

Once transformed into *Eschereschia coli *bacteria, clones from the G19833 library were picked by an automated robotic colony picker to a total of 10,000 clones (half from each library). A total of 384 individual clones were sized and sequenced from both ends at RIKEN to determine their insert size and quality before sending a total of 9,984 clones (approximately half the library) for sequencing at the Washington State University sequencing center in St. Louis, Missouri. A total of 26 plates were sequenced from glycerol stocks on automated capillary DNA sequencers (ABI 3730× from Applied Biosystems). Sequencing was performed using the M13-21 primer (5'-TGTAAAACGACGGCCAGT-3') to evaluate the clones from the 5' end of the full-length cDNA inserts. Although we made two libraries (one Andean from G19833 and one Mesoamerican from BAT477) we only sequenced from the first of these given funding constraints and given the relative importance of G19833 which is being sequenced by a whole genome shotgun approach (S. Jackson, pers. communication).

### Data assembly

Sequence reads were trimmed for low quality and vector contamination. This was accomplished for each full length EST sequence using Phred software [44] to eliminate low quality regions with more than one N in a 100 bases or a stretch of bases with Phred quality score < 20. These were generally found at the 3'end of the sequences and were discarded. Vector sequences, meanwhile, were eliminated by TrimVector (Sequencher, Ann Arbor, MI) using a database of vectors found at NCBI followed by manual confirmation with local cleaning using the software program Geneious (Biomatters Ltd, Auckland, New Zealand) to check for poor sequences. Poly-N stretches were masked and small insert clones (< 100) or hits to *E. coli *sequences disregarded. Poly-A tails were identified and trimmed at their adjacent base if followed by vector sequences. The software program BLAST2GO from [45], and which is a part of Blast 2.2.23 [46], was used to remove any hits to ribosomal, chloroplast and mitochondrial sequences along with non-plant hits. The software CAP3 from [47] with default parameters (gap penalty factor, N > 6; segment pair score cutoff, N > 40, overlap length score cutoff N > 80, etc) was used to assemble the sequences from the full-length cDNA clones allowing us to assemble the newly-created and cleaned full-length ESTs into contigs and unigenes. Comparisons were made between the distribution of cleaned sequences in the full-length cDNA library compared to those of Ramírez et al. [12] and Thibivilliers et al. [14].

### Gene annotation and comparative genomics

BLAST2GO was again used with the assembly of EST sequences to determine the top-hit distribution of each unigene and contig from the full-length cDNA database. Searches were made against corresponding non-redundant (nr) database with blastx against all higher plant proteins. The E-value and positive alignment length distributions were then determined with a high-scoring segment pair (HSP) cutoff of 33 and an E-value threshold of 1E^-3^. Gene ontologies (GO) were assigned based on Harris et al. [48] and by evaluating against Uniprot, TAIR, GR-protein and Ecosys databases. Genes were then evaluated for their likely molecular cellular function, cellular localization and involvement at four gene ontology levels. Gene annotation for the 5'untranslated region (UTR) and open-reading frame (ORF) border were made by comparing the BLAST2GO report for the beginning of known proteins and matching these with the start codon for each unigene using an E-value threshold for hits of 1E^-6 ^up to a cutoff of 1E^-55 ^Comparisons were also made to sequences from Ramírez et al. [12] and Thibivilliers et al. [14] and with the annotation of the soybean (*G. max*) genome and soybean ESTs to determine intron/exon boundaries and to confirm probable start codons. In addition, KEGG annotation was also used to determine KO ontologies and to determine the position of the unigenes and singletons form the full assembly described above in various biochemical pathways and directed acyclic graphing (DAG) was used to determine the gene relationships. Finally, simple sequence repeats were identified in the full-length sequences based on their locations within the 5'UTR or ORF using first the software program RepeatFinder [49] and then more definitively SciRoKO [33].

## Results

Two full-length cDNA libraries were successfully constructed using the CAP-trapper technology, one for the drought tolerant Mesoamerican genotypes BAT477 and one for the acid-soil tolerant Andean genotype G19833. The libraries were based on totals of 3.789 mg and 4.258 mg of high-quality total RNA obtained from the six different irrigation × soil treatments for these two genotypes, respectively, which was sufficient for the highly complex process of full-length mRNA selection from polyA mRNAs. For the libraries, a total of 20,000 clones were selected robotically (half from each genotype) and in preliminary sequencing of 5'and 3'ends the clones of both libraries (a 384 well plate each) were shown to average 1.5 kb in length and to have non-chimeric sequences mostly with poly A tails at their 3'end. Additional file 3 shows the distribution of the initial clones in terms of total length.

Following this initial testing, nearly 10,000 clones were sequenced from the Andean G19833 library. The sequences were all from 5'ends with an average read length of 850 nucleotides (nt). Of these, 9626 surpassed the initial threshold of low number of N's and had an average read length of 781 nt. Upon vector trimming the average insert length was 564 nt and the number of cleaned sequences after trimming and low quality sequence elimination was 7039. Sequences were submitted to GenBank (entry numbers JK037067-JK044145) and consisted of both singletons and independent clones within each contig. Table 1 shows the comparison in success rate of sequencing in three recent EST efforts for common bean, comparing the full-length cDNA library to non-full length libraries sequences by Ramírez et al. [12] and Thibivilliers et al. [14]. Success rate of sequencing for this library (71%) was comparable to the 75% of Ramírez et al. [12]. After assembly of all the full-length cDNA clones, a total of 4219 unigenes were identified. These consisted of 1238 singletons (29.3% of unigenes and 17.5% of sequences) and 2981 contigs (70.7% of unigenes and 42.1% of sequences) assembled with CAP3. On average the number of sequences within a contig was 3.3 with an average length of the contigs being 678 nt. Meanwhile, the average length of the singletons was 568 nt. The large number of singletons and the high number of contigs relative to the number of sequences indicated low redundancy for the library. This was not surprising given that the plants for the RNA extraction had been grown in three different soil treatments and under both drought-stress and irrigated conditions and that whole plants (above and below ground parts) were harvested at seven and three timepoints for the cDNA preparations, respectively.

**Table 1:** Comparison of major EST sequencing efforts in common bean.

Sequence read	Full-length	Ramírez	**Tibivilliers**^**1**^
Clones	9,984	21,096	20,736
Sequence reads (after LQ & vector trimming)	7,079	15,781	37,919
Singletons	1,238	5,703	3,544
Contigs	2,981	2,266	7,510
Unigenes	4,219	7,969	10,581
Proportion of unigenes per sequence (%)	59.6%	50.5%	27.9%
Average EST length (nt)	563.8	606.2	656.4
Average contig length (nt)	677.9	606.2	1024.2
Average singleton length (nt)	568.3	594.7	691.7

Two EST sequencing efforts are compared to the ESTs generated for the full-length cDNA libraries made in this study

^1 ^clones from Thibivilliers et al. (2009) sequenced from both ends of the insert while those of Ramirez and this study were 5'end sequenced.

**Figure 1:**
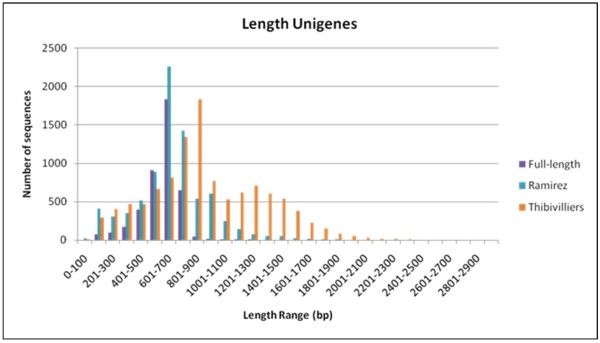
**Comparison of unigene length among the full-length cDNA libraries and two other EST sequencing efforts**. The full-length cDNA clones were sequenes from from the 5'end as were the clones from Ramírez et al. (2005) while those of Thibivilliers et al. (2009) were sequenced from both 5 'and 3' ends.

The full-length unigenes were compared to other EST sequencing efforts of Ramírez et al. [12] and Thibivilliers et al. [14] in common bean for the length of the unigenes identified which was moderate and well-distributed for the full length library compared to intermediate lengths (606 nt) in the first study but higher averages for the second study (1024 nt) where unigenes were made up of both 5' and 3' sequences for each clone. This created scaffolds or full-length genes for the Thibivilliers et al. [14] library which was evidenced by the bimodal peak of longer unigenes to the right of Figure 1 for those unigenes. In neither the present study or for Ramírez et al. [12] were 3' end sequences included in the construction of the unigene set, explaining the shorter unigene lengths.

**Figure 2:**
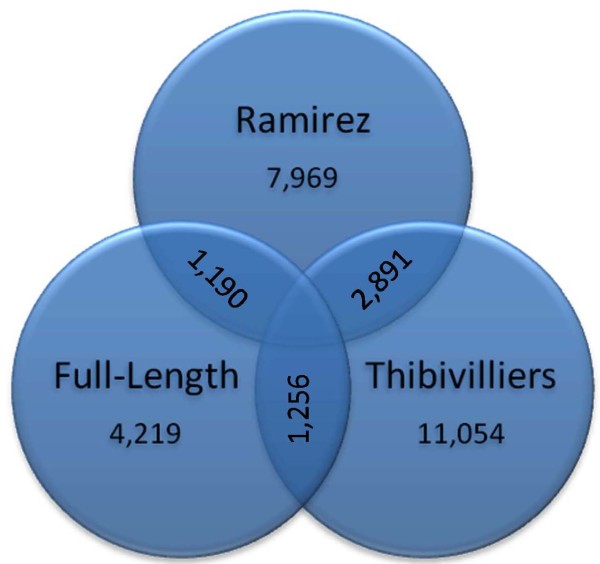
**Overlap in homology at E-threshold of 1E^-100 ^for full length cDNA library and two previous EST sequencing efforts**. The previous EST sequencing were from Ramírez et al. (2005) and Thibivilliers et al. (2009).

**Figure 3:**
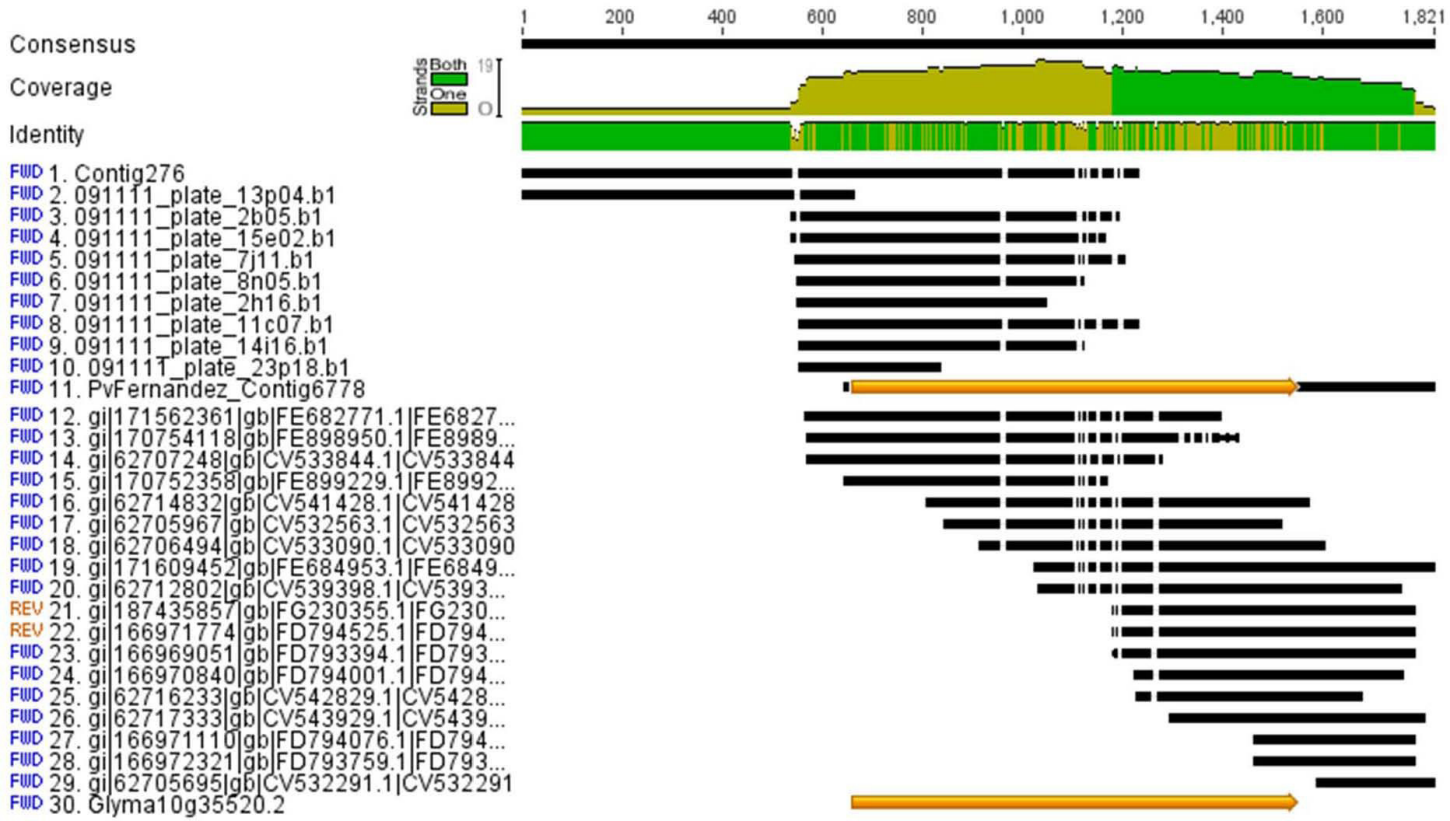
**Example of alternate splicing and 5'end location of an EST for aquaporin from the full-length library**. Comparison of non-full-length ESTs (below line 11) to full-length ESTs (above line 11) for a gene with high homology to GenBank accession ABU94631 encoding a putative aquaporin PIP2;3 from *Phaseolus vulgaris*. The last arrow at the bottom of the figure shows the homology to the equivalent ortholog in the soybean genome.

In comparisons of the full-length cDNA library assembly with EST sequencing efforts of Ramírez et al. [12] and Thibivilliers et al. [14] we found that about half of the sequences were novel and a significant number represented the 5' portions of previously discovered genes from other EST efforts as shown by the diagrams in Figure 2 and 3. In this study, a total of 3029 of the full-length cDNA unigenes were novel compared to those of Ramírez et al. [12] while 1190 were similar. For the work of Thibivilliers et al. [14], 1256 were similar and 2963 were novel. Among the studies of Ramírez et al. [12] and Thibivilliers et al. [14] there was an overlap of only 2891 similar unigenes and EST sequences. In the case of this analysis of re-assembly of Thibivilliers et al. [14] ESTs gave 11054 unigenes compared to what they report of 10581 unigenes. Meanwhile, almost 78% of the individual full-length EST unigenes had homology to a soybean gene of known or unknown function (3280 out of 4219 contig or singleton sequences) and only 391 were bean-specific based on having no hits to any of the plant databases. Homologies to genes from medicago (1221 unigenes, 28.9%) or arabidopsis (147 unigenes, 3.5%) were lower based on blastn searches using a 1E^-30 ^threshold.

In our functional analysis of the unigenes discovered in the full-length cDNA library, we found that BLAST2GO found a range of hits from our low threshold of 1 × 10^-10 ^up to 1 × 10^-175 ^and similarity values ranging from 40 to 100% alignment within a range of nucleotide windows. Top species hit for the full-length unigenes was with soybean (over 1,500 unigenes) and then grape (over 500 hits). These were followed by 250 to 400 hits each with medicago, poplar and castor bean.

**Figure 4:**
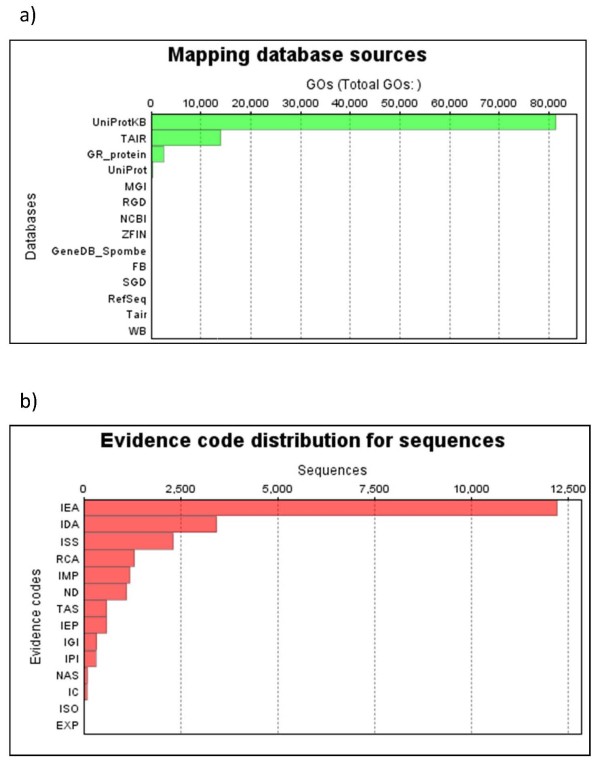
**Mapping databases with (a) top hits for gene ontology (GO) and (b) gene ontology evidence code distribution for blastx hits in terms of numbers of GO terms or sequences (y-axes), respectively**. Based on the full collection of unigenes from the full-length cDNA library sequencing project.

Only 100 highest hits were directly with common bean genes. These numbers of hits reflect both the similarity of the species, especially in the case of common bean-soybean, and the number of unigenes that exist in GenBank under the non-redundant UniProtKB or TAIR databases which were most frequently used for mapping of unigene ontology (Figure 4). The most frequently expressed genes were generally housekeeping genes but did reflect the abiotic stress conditions for the plants from which the full-length library was made and sequenced (Additional file 4) including the propensity of finding aquaporins which are useful for water uptake in cells under osmotic stress (Figure 3). Indeed contigs 147 and 1001 represented TIP and MIP type aquaporins containing 19 and 20 ESTs, respectively.

In the gene ontology analysis (Table 2), various levels were evaluated and at level 2 for biological processes a quarter of the genes each fell into either cellular (28.4%) or metabolic (26.7%) categories. For molecular functions the genes were almost evenly divided between binding (42.5%) and catalytic functions (37.8%), followed by transporter activity (5.8%). Cellular components were divided among cell, organelle and to a lesser extent cell membrane and extracellular regions. These values were different in diversity compared to values for a recent set of root ESTs made in our laboratory [15]. For example response in the full-length library there were higher percentages of genes for biological regulation, developmental processes and response to stimuli. Since the full-length library was made from aerial as well as below-ground tissue differences of this nature would be expected.

**Table 2:** Differences in categorization of the unigenes from the full length cDNA library compared to two other recent EST libraries of common bean.

Gene Ontology	Full-length library (this study)	%	Root library (Blair et al., in press)	%
*Biological process^2^*				
Biological regulation	524	8.9	204	6.5
Carbon utilization	13	0.2	3	0.1
Cell proliferation	0	0.0	4	0.1
Cellular component organization	225	3.8	119	3.8
Cellular processes	1669	28.4	920	29.4
Cellular component biogenesis	98	1.7	80	2.6
Developmental process	307	5.2	136	4.3
Death	22	0.4	7	0.2
Immune system process	48	0.8	11	0.4
Localization	374	6.4	212	6.8
Metabolic process	1567	26.7	871	27.8
Multi-organism process	0	0.0	54	1.7
Multi-cellular organismal process	251	4.3	115	3.7
Nitrogen utilization	0	0.0	4	0.1
Pigmentation	0	0.0	1	0.0
Rhythmic processes	0	0.0	2	0.1
Reproduction	141	2.4	72	2.3
Response to stimulus	638	10.9	310	9.9
Sulfur utilization	0	0.0	4	0.1
*Molecular function^2^*				
Antioxidant activity	34	0.9	28	1.3
Binding	1656	42.5	841	40.4
Catalytic activity	1472	37.8	704	33.8
Electron carrier activity	108	2.8	61	2.9
Enzyme regulator activity	34	0.9	21	1.0
Metallochaperone activity	2	0.1	1	0.0
Molecular transducer activity	53	1.4	15	0.7
Nutrient reservoir activity	10	0.3	5	0.2
Protein tag	2	0.1	0	0.0
Structural molecular activity	101	2.6	193	9.3
Translation regulator activity	51	1.3	40	1.9
Transporter activity	227	5.8	113	5.4
Transcription regulator activity	148	3.8	60	2.9
*Cellular component^3^*				
Apoplast	95	1.9	58	2.0
Cell part	2176	44.6	1200	40.9
Extracellular region	0	0.0	2	0.1
Organelle part	404	8.3	364	12.4
Organelle - membrane bounded	1500	30.7	779	26.5
Organelle - non membrane bound	181	3.7	265	9.0
Protein complex	271	5.6	134	4.6
Protein-DNA complex	17	0.3	23	0.8
Vesicle	236	4.8	112	3.8

Gene ontology according to biological processes, molecular function and cellular compartmentalization.

^1 ^refers to number of unigenes in each case for high phosphorus (HP) or low phosphorus (LP) root libraries.

^2 ^performed at level 2 for greater details of biological process and molecular function.

^3 ^performed at level 3 for greater details of cellular component placement.

Additional differences were observed for the analysis of the full-length cDNA library here and the libraries from Ramírez et al. [12] for leaf tissue and for that of Thibivilliers et al. [14]. This shows the high degree of normalization of the full-length cDNA library given its mix of root, shoot and leaf tissues from various water treatments and soil growth conditions. Response to abiotic stress, generalized stress, chemical stimuli, cellular as well as macro-molecular metabolic, primary metabolic, oxidation/reduction and catabolic processes were all important in the third level of biological processes (data not shown). The frequency of aquaporins in the library may reflect the adaptation to drought stress in half the tissue sources used for full-length cDNA library construction.

**Figure 5:**
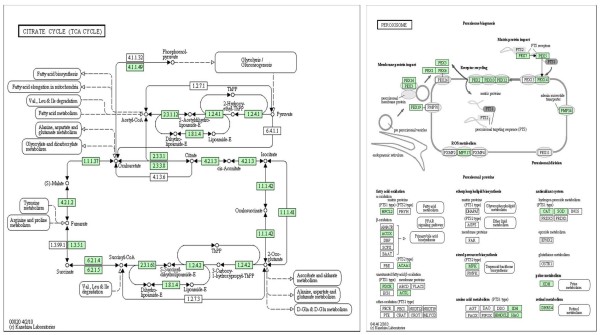
**Example of KEGG pathways found for the full-length cDNA clone ESTs**. The examples of Citrate Cycle and Peroxisome function are given with genes in green represented by full-length cDNA clones from the newly constructed library.

By comparing the GO annotation of unigenes from the full-length cDNA library with the Kyoto Encyclopedia of Genes and Genomes (KEGG) we were able to come up with a KO annotation with the major categories being in decreasing order: translation, energy metabolism, carbohydrate metabolism, transcription, co-factor metabolism, replication and repair, amino acid metabolism, signaling mechanisms as well as unclassified genes. Examples of KEGG pathways for the Citrate Cycle and Peroxisome function are shown in Figure 5 where genes represented in the full-length cDNA library are highlighted. The Peroxisome pathway is given as an example of a pathway that is influenced by various stresses such as the ones sampled in the library construction (drought, low P and high Al effects on roots and above-ground tissues) while the Citrate cycle, while influenced by these stresses, is full of more constitutive genes.

In terms of comparisons of the two genotypes used to create the full-length cDNA libraries (G19833 versus BAT477) this was limited by the EST sequencing which only in the initial stage used both libraries. Furthermore, SNP identification would have required 3'sequencing given that the individual tags for the genotypes were on the 3' end. Therefore it was not possible to carry out the single nucleotide polymorphism analysis comparison of the two genotypes we originally had planned, although we were able to identify a large number of simple sequences repeats using two different search engines with tri-nucleotide repeats more common than di-nucleotide repeats (Table 3). Marker identification in terms of SSRs varied with RepeatFinder identifying only 175 in total (2.5% of ESTs) but SciRoKo finding a total of 1,932 (24.3% of ESTs).

**Table 3:** Simple sequence repeats found by two software programs in the unigene set of full-length cDNA sequences.

SSR class	Repeat Finder	%	SciRoKo w/o mono-nt	%	SciRoKo w/mono-nt	%
Mono-nt	0	0	0	0.0	468	24.2
Di-nt	29	16.6	322	22.0	322	16.7
Tri-nt	89	50.9	562	38.4	562	29.1
Tetra-nt	28	16.0	104	7.1	104	5.4
Penta-nt	13	7.4	193	13.2	193	10.0
Hexa-nt	16	9.1	283	19.3	283	14.6

Total	175	100	1464	100	1932	100

Comparison of RepeatFinder and SciRoKO software shown in number of repeats found and percentage of total for each SSR class from mono-nucleotide (nt) repeat to hexa-nt repeat.

## Discussion

The major success of this research was the construction of two full-length cDNA libraries from two important common bean genotypes grown under three types of abiotic stress and the preliminary sequencing of the libraries with approximately 7,000 ESTs, with an average of 564 nucleotides in length. The success rate of the EST sequencing effort is equivalent to that of Ramirez et al. [12] but slightly lower than that of Thivibilliers et al. [14] which is to be expected given the library movement from RIKEN to Univ. of Washington for sequencing. The high rate of unigenes per HQ sequence is fairly unique among cDNA libraries established for common bean to date and represents the broad representation of tissues used to create the full-length cDNA library and its predominant coverage of the 5'end of the ESTs.

A similar approach was taken by Umezawa et al. [28] when constructing a full-length cDNA library for soybean in which they used a total of seven stresses (drought, salt, freezing, low temperature, P starvation, flooding and nematodes) along with three specialized tissue types (flower buds, nodules and developing seeds) for their RNA extracts. We used a similar strategy and sampled all organs from both below ground and above ground parts up to 35 days of growth under the important common bean stresses of low phosphorus, aluminum toxicity and drought [2,20]. We did not include developing seeds or pods because of the limitations of growing plants under cylinder pot culture where reproductive stage tissue is of lower quality than vegetative stage tissue. We sampled roots extensively by careful separation from soil.

In addition to the library construction itself, the EST sequencing now brings the total of ESTs in common bean to almost 120,000 sequences. Considering that most previous ESTs were in the range of 500 to 600 nt and were from regular cDNA libraries that only represented specific tissues and partial sequences starting from random points in the gene sequences (usually in the ORF) this work had the advantage of providing more diverse ESTs for the species. In addition we obtained many longer EST reads (from 700 to 900 nt long) that mostly represented the 5'UTR plus the often most crucial start codon and first exon of the ORFs, rather than the 3' ends typical of regular EST projects. This is a true benefit of full-length cDNA library construction and allowed us to conduct proper gene modeling of exons and introns when cDNA sequences were compared against genomic sequences of soybean for example. It is expected that the full-length cDNA sequences will be useful for annotation of the genome. Another advantage of our efforts in EST sequencing was derived from the fact that the multiple-tissue sampling that we did was found to increase the proportion of unigenes in certain gene ontology categories compared to other libraries. For example, it was notable that the number of total unigenes of the full-length library was almost two thirds the number of Ramírez et al. [12] despite half the sequencing effort, showing the success of mixed tissue libraries and full-length cDNA construction as a strategy for gene discovery.

This advantage of the full-length cDNA library and cap-trapping developed at RIKEN is based on the chemical introduction of a biotin group into the diol residue of the cap structure of eukaryotic mRNA. This step is followed by digestion by RNase I, a ribonuclease that can cleave single-stranded RNA at any site and by selection of full-length cDNA. The libraries produced by this method contained a very high proportion of full-length cDNAs and produced an excellent yield without involving PCR amplification which could introduce bias in the representativeness of the clones. Carninci et al. [22] also found that by introducing the disaccharide trehalose to the reverse transcriptase reaction at the high reaction temperature of 60°C resulted in the synthesis of even longer full-length cDNAs, and higher representation of long full-length cDNAs in the library. In summary, this method of selecting full-length cDNAs by biotinylation of the mRNA cap and streptavidin capture followed by the use of the trehalose-thermostabilized reverse transcriptase, made it possible to prepare longer full-length cDNAs, and at the same time to remove non-full-length cDNAs [24,25]. This method has shown itself to be ideal for construction of high-content full-length cDNA libraries for various crops, to analyze domain order and determine start codons. We are lucky now to have common bean be the second legume to have one of these libraries available after soybean [28].

Recently, emphasis on EST sequencing has waned due to the advent of next generation sequencing techniques that can quickly dissect a transcriptome. However, full length cDNA sequences will remain useful in the discovery of alternative splice sites and for unraveling paralogs within gene families. For common bean full length cDNAs this retains even more relevance as it is a basal member of the *Phaseoleae *tribe which contains other important species such as soybean, cowpea and pigeonpea [1.50,[51]]. The comparisons of ESTs in diploid common bean and ancestral tetraploid soybean are likely to be important so as to discover which exact copies of genes are orthologs and in that manner make use of each species as a model for the other. Currently the number of ESTs within the Phaseoleae tribe is the highest among the legumes (2.2 M reads) compared to other legume tribes (Cicereae, Dabergieae, Fabeae, Galegeae, Loteae and Trifolieae) which only have from 30 to 300 thousand each. Indeed, the tropical genera *Cajanus*, *Glycine*, *Phaseolus *and *Vigna *all represent economically important food or industrial crops while in the cool season legumes much of the EST sequencing has been done in model or forage species of arguably lesser importance [50]. Finally, the increasing number of sequences in the *Phaseoleae *reflects a bias towards the Papilionoideae legumes compared to the Caesilpinoids and Mimosid families with far fewer ESTs each.

The number of sequences available in related clades is increasingly correlated with the ease of marker development in any one member of each plant species or clade. We have found the full-length cDNA library useful for finding the 5'UTR and start codons of various genes from pathways that are important to legumes, such as abiotic stress response or nutrient accumulation. In this work we initiated the search for a new set of EST-SSR with the thought that given the high proportion of 5'UTR sequences in the ESTs we have obtained, there would be new classes and perhaps more polymorphic SSRs. So far we have applied two programs finding from between 175 and 1,400 SSRs and are contemplating the use of other software for a more complete analysis before embarking on primer design. ESTs are known to be rich sources of SSRs [52]. The difference in EST-SSR frequency found between RepeatFinder and SciRoKo could be due to the algorithm in SSR identification especially as the second software found mono-nucleotide repeats (without these only 1,464 SSRs were identified equivalent to 18.1% of ESTs) while the first software found 2.5% of ESTs to have SSRs. In other future work, we plan to take advantage of the two libraries we have made which are tagged at the 3' end with two different tags which distinguish each of the genotypes we used for library constructions. Our plan it to sequence from the 3'end of the full-length clones already sequenced to construct scaffolds for each gene and then to compare the populations of cDNA clones from each genotypes for single nucleotide polymorphism (SNPs). We are beginning to evaluate assemblies of full-length cDNA sequences with regular EST sequences from common bean and related species to identify possible SNPs through comparison of Andean versus Mesoamerican or *P. vulgaris *versus *P. coccineus *assembled ESTs. This is possible since many of the EST libraries made so far for common bean represent a range of varieties from Andean snap beans (Early Gallatin) to Mesoamerican dry beans (Negro Jamapa).

In addition we will soon have the genomic sequences of the Andean landrace G19833 genotype and the Mesoamerican breeding line BAT93 genotype for these comparisons. Our plan is to sequence additional 5'and 3'ESTs from the two libraries in a forthcoming paper so as to determine the genotype of each clone and use these for bioinformatics analysis of single nucleotide polymorphisms in the comparison of BAT477 and G19833 with each other and with the sequenced BAT93 genome. The other aspect of genome annotation that we are interested in and which might be readily assisted by more sequencing of these libraries is in the uncovering of multi-gene families by distinguishing them at their 5'ends and in determining promoter sequences that are with in expressed transcripts. In this sense the aquaporin we identified as an example of alternate splicing is interesting for further characterization. The study of how completely the full-length clones covered parts of individual KEGG pathways was also interesting because it gave evidence of pathways that are turned on during stress generally (the perixosime pathway for example) or that have been shown to be both constitutive an related to specific stresses (such as the citrate cycle pathway which is important for generating organic acids during aluminum stress). Another activity will be to determine how representative the full-length cDNA clones are of all genes predicted to be expressed from the whole genome sequence.

## Conclusions

The value of full length cDNA libraries is in their utility for the correct annotation of genomic sequences and functional analysis of genes because of their representativeness of the 5"UTR and full ORF of most genes, unlike other cDNA cloning or EST sequencing efforts (Seki et al. 2002). When seqecuenced from both 5'and 3'end they can be used to create physical scaffolds that definitively determine transcript length. Since they are each unique clones, they are more useful for determining alternate splicing sites. This is the second set of full-length cDNA libraries made for a legume and one of the first outside of cereals, cassava or arabidopsis. As such it is important to make use of the information gathered from this library for marker development and genome characterization useful for plant breeding.

Finally, the present full-length cDNA library adds substantially to the number of unique EST sequences available for the common bean genome and especially provides 5'end sequences that are more unique and useful for gene identification. These EST tags should be useful for functional gene annotation, analysis of splice site variants and intron/exon determination, and evaluation of gene homologies or KEGG pathway confirmation especially as future whole-genome sequences become available.

## Authors' contributions

MWB obtained the funding and drafted the paper. MI and MWB planned the experiments along with DM. ACF and SA performed the bioinformatics analysis. ACF prepared figures and edited tables. DM carried out the RNA extractions. MS and SS and constructed full-length cDNA library and did preliminary sequencing and analysis. All authors read and approved the manuscript.

## Supplementary Material

Additional file 1 **Table showing the soil and irrigation conditions used in the experiments to develop the full length library for tissue from the genotype G19833**. The experiment included two irrigation (well-watered vs. terminal drought) treatments × three soils (Darién, Palmira, Santander de Quilichao). A similar experiment was performed with BAT477 as with G19833. Click here for file

Additional file 2 **Figure showing the design of the experiment used to collect tissue for the full-length library construction of G19833 tissues**. Tissue collection was made at the indicated time points from root and shoot tissue (including seedlings, growing tips, leaves, stems, shoots, flowers, small pods and roots) of soil-cylinder grown plants shown to the right of the diagram in a photographic insert of three soil tubes under water stress. A similar experiment was performed with BAT477 as with G19833. Click here for file

Additional file 3 **Figure showing the distribution of (a) E-value and (b) sequence similarity distributions against the GenBank database**. Based on the full collection of unigenes from the full-length cDNA library sequencing project. Click here for file

Additional file 4 **Table showing the 40 top-most genes in frequency (EST counts) expressed in the full-length library**. Gene homology for each of the contigs shown. Click here for file
