# Supplementary material for: Construction and EST sequencing of full-length, drought stress cDNA libraries for common beans (Phaseolus vulgaris L.)
Source: BMC Plant Biol. 2011 Nov 25;11:171. doi: 10.1186/1471-2229-11-171 (PMC3240127; doi:10.1186/1471-2229-11-171)
Supplement: Additional file 1 — Table showing the soil and irrigation conditions used in the experiments to develop the full length library for tissue from the genotype G19833. The experiment included two irrigation (well-watered vs. terminal drought) treatments × three soils (Darién, Palmira, Santander de Quilichao). A similar experiment was performed with BAT477 as with G19833. [file 1471-2229-11-171-S1.DOCX]

**Additional File 1. Table showing the soil and irrigation conditions used in the experiments to develop the full length library for tissue from the genotype G19833.** The experiment included two irrigation (well-watered vs. terminal drought) treatments x three soils (Darién, Palmira, Santander de Quilichao). A similar experiment was performed with BAT477 as with G19833.

| Expt. No | Irrigation treatments | Soil treatments  (chemical/physical properties) |
| --- | --- | --- |
| 1 | Well-watered (irrigation) | Darién (low P content) |
| 2 |  | Palmira (highly compacted) |
| 3 |  | Santander de Quilichao (high Al content) |
| 4 | Drought-stressed (terminal drought) | Darién (low P content) |
| 5 |  | Palmira (highly compacted) |
| 6 |  | Santander de Quilichao (high Al content) |
