# Supplementary material for: Construction and EST sequencing of full-length, drought stress cDNA libraries for common beans (Phaseolus vulgaris L.)
Source: BMC Plant Biol. 2011 Nov 25;11:171. doi: 10.1186/1471-2229-11-171 (PMC3240127; doi:10.1186/1471-2229-11-171)
Supplement: Additional file 2 — Figure showing the design of the experiment used to collect tissue for the full-length library construction of G19833 tissues. Tissue collection was made at the indicated time points from root and shoot tissue (including seedlings, growing tips, leaves, stems, shoots, flowers, small pods and roots) of soil-cylinder grown plants shown to the right of the diagram in a photographic insert of three soil tubes under water stress. A similar experiment was performed with BAT477 as with G19833. [file 1471-2229-11-171-S2.DOCX]

**Additional File 4. Table showing the 40 top-most genes in frequency (EST counts) expressed in the full-length library.** Gene homology for each of the contigs shown.

| **Contig ID** | **Count** | **Gene homology** |
| --- | --- | --- |
| Contig1018 | 48 | methionine 5-methyltetrahydrotriglutamate-homocysteine |
| Contig145 | 38 | Ubiquitin |
| Contig323 | 29 | peroxisomal glycolate oxidase |
| Contig903 | 27 | translationally controlled tumor protein |
| Contig1117 | 24 | chaperone protein |
| Contig991 | 21 | secretory peroxidase |
| Contig147 | 20 | tonoplast intrinsic protein |
| Contig165 | 20 | cysteine protease |
| Contig1001 | 19 | MIP PIP subfamily |
| Contig152 | 18 | fructose bisphosphate aldolase |
| Contig1022 | 17 | elongation factor 2 |
| Contig204 | 17 | asparagine synthetase |
| Contig84 | 17 | pathogenesis related protein |
| Contig504 | 16 | type 2 metallothionein |
| Contig855 | 16 | lipoxygenase l-5 |
| Contig564 | 15 | sucrose synthase |
| Contig576 | 15 | plasma membrane intrinsic protein |
| Contig72 | 15 | lhca2 protein |
| Contig900 | 14 | isoflavone synthase 1 |
| Contig189 | 13 | elongation factor 1- |
| Contig245 | 13 | Cyclophilin |
| Contig980 | 13 | mip pip subfamily |
| Contig1127 | 12 | glycine-rich RNA-binding protein |
| Contig527 | 12 | Catalase |
| Contig69 | 12 | mip tip subfamily |
| Contig1033 | 11 | s-adenosylmethionine synthetase |
| Contig107 | 11 | ECT2 protein binding |
| Contig255 | 11 | unknown protein |
| Contig688 | 11 | phosphoglycerate kinase |
| Contig776 | 11 | lipoxygenase l-5 |
| Contig1084 | 10 | Unknown protein |
| Contig1143 | 10 | s-adenosylmethionine decarboxylase |
| Contig1217 | 10 | beta chain protein |
| Contig131 | 10 | proline-rich protein |
| Contig144 | 10 | burp domain-containing protein |
| Contig200 | 10 | unknown protein |
| Contig483 | 10 | cysteine protease |
| Contig49 | 10 | unknown protein |
| Contig573 | 10 | vacuolar h+-atpase catalytic subunit |
| Contig665 | 10 | type 1 metallothionein |
