# Supplementary material for: Construction and EST sequencing of full-length, drought stress cDNA libraries for common beans (Phaseolus vulgaris L.)
Source: BMC Plant Biol. 2011 Nov 25;11:171. doi: 10.1186/1471-2229-11-171 (PMC3240127; doi:10.1186/1471-2229-11-171)

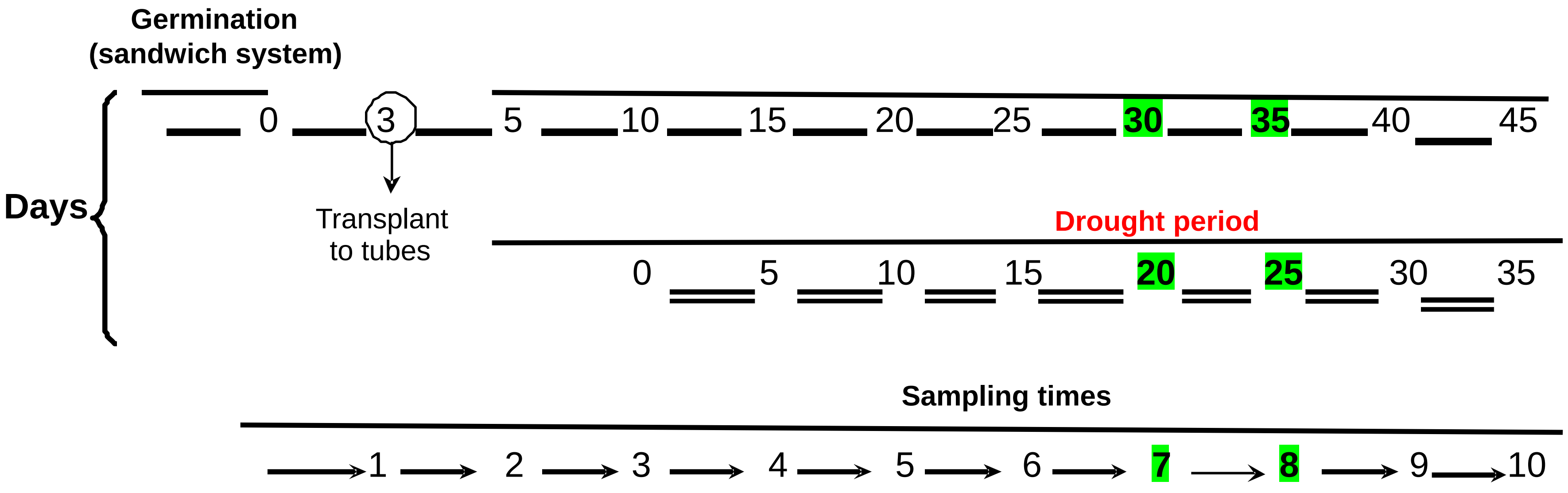

Legend for time points

Days after the establishment of the experiment (germination).

Days under drought stress.

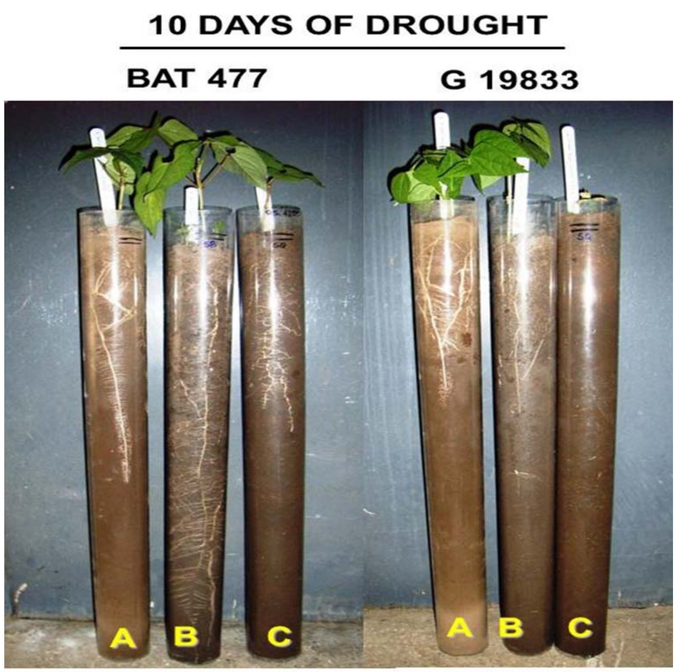

Supplement: Additional file 3 — Figure showing the distribution of (a) E-value and (b) sequence similarity distributions against the GenBank database. Based on the full collection of unigenes from the full-length cDNA library sequencing project. [file 1471-2229-11-171-S3.PDF]
