# Supplementary figures and images for: Construction and EST sequencing of full-length, drought stress cDNA libraries for common beans (Phaseolus vulgaris L.)
Source: BMC Plant Biol. 2011 Nov 25;11:171. doi: 10.1186/1471-2229-11-171 (PMC3240127; doi:10.1186/1471-2229-11-171)

a)

### E-value distribution

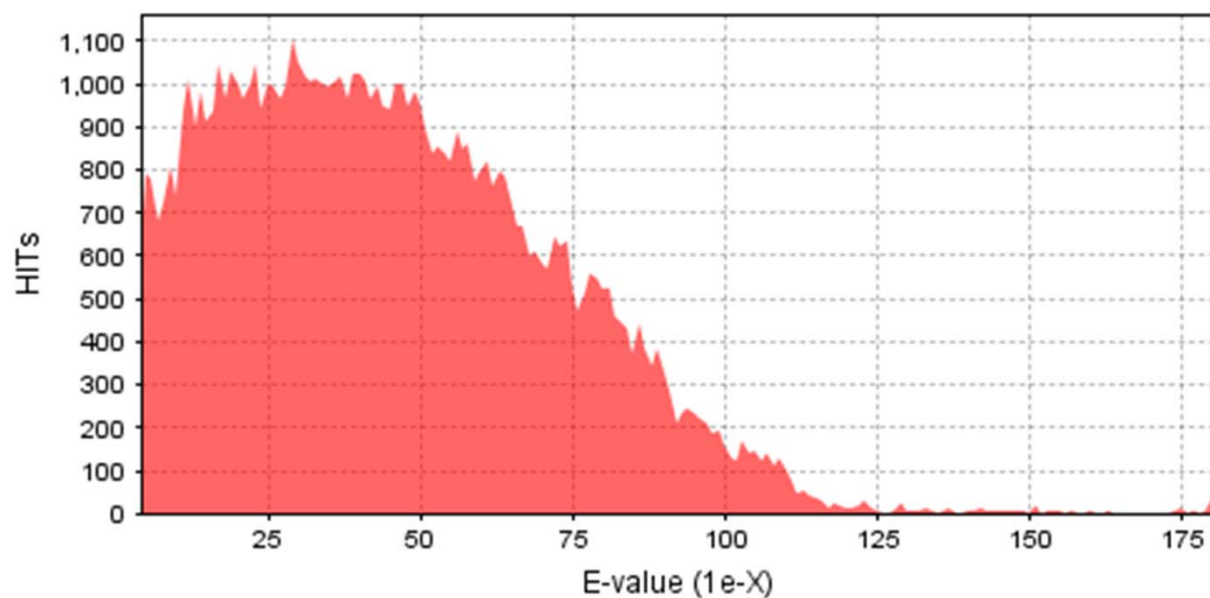

b)

### Sequence similarity distribution

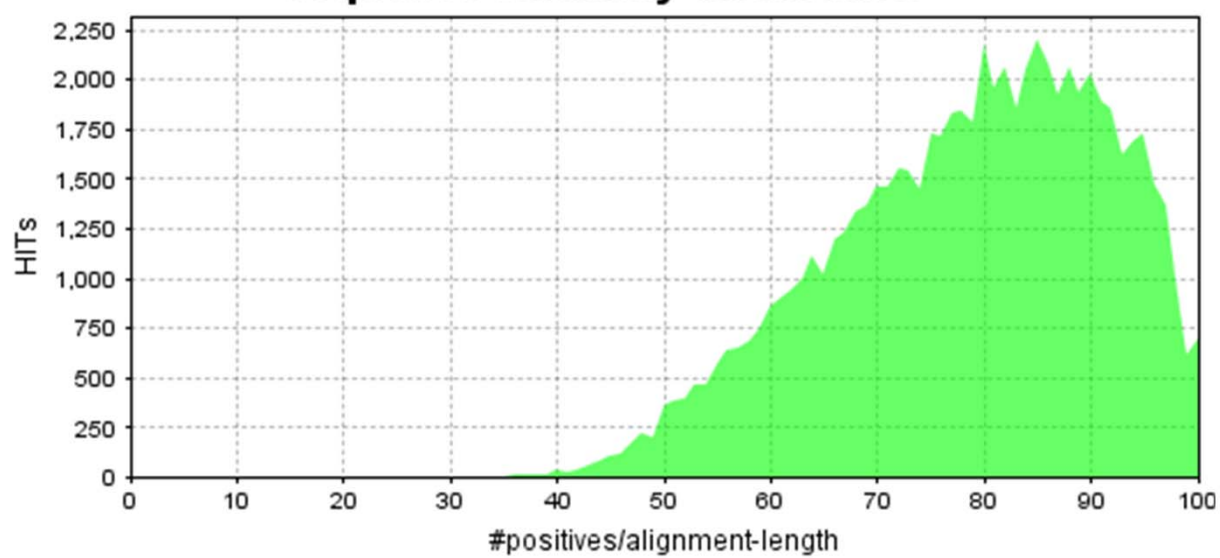

Supplement: Additional file 4 — Table showing the 40 top-most genes in frequency (EST counts) expressed in the full-length library. Gene homology for each of the contigs shown. [file 1471-2229-11-171-S4.PDF]
